# “The Women’s PrEP Project,” a Clinic-Based, Socio-Structural Intervention to Improve the Provision of Preexposure Prophylaxis for Cisgender Women: Interrupted Time Series Pilot

**DOI:** 10.2196/80653

**Published:** 2025-12-03

**Authors:** Rachel K Scott, Shawnika J Hull, Deanna Kerrigan, Yan Wang, Mandi Pratt-Chapman, Tara Mathias-Prabhu, Naquia Unwala, Marisa Sadauskas, Bat-zion Hose, Marjanna Smith, Patricia Moriarty, Tranessa Hanson, Ariam Tedla, Pamela Lotke, Peggy Ye, Hannah Arem

**Affiliations:** 1 Department of Obstetrics and Gynecology, School of Medicine, Georgetown University, Washington, DC, United States; 2 Women's and Children's Research Network, MedStar Health Research Institute, MedStar Health, 110 Irving St NW, East Building 5108, Washington, DC, 20010, United States, 1 202-877-7541; 3 Women's and Infants' Services Department, MedStar Washington Hospital Center, Washington, DC, United States; 4 Department of Communication, School of Communication & Information, Rutgers, The State University of New Jersey, New Brunswick, NJ, United States; 5 Department of Prevention and Community Health, Milken Institute School of Public Health, George Washington University, Washington, DC, United States; 6 Center for Patient-Centered Initiatives and Health Equity, Cancer Center, George Washington University, Washington, DC, United States; 7 School of Medicine, Saint Louis University, Saint Louis, MO, United States; 8 MedStar Health National Center for Human Factors in Healthcare, Medstar Health Research Institute, Washington, DC, United States; 9 Implementation Science, Healthcare Delivery Research Program, MedStar Health Research Institute, Washington, DC, United States

**Keywords:** preexposure prophylaxis, female, primary prevention, education, HIV infections

## Abstract

**Background:**

Cisgender women account for 23% of new HIV diagnoses in the United States, but there are significant socio-structural barriers to engagement and retention in the preexposure prophylaxis (PrEP) cascade, particularly for women of color.

**Objective:**

In response to the lack of evidence-based interventions to improve PrEP initiation, adherence, and persistence among women in the United States, we developed and piloted a clinic-based, socio-structural intervention to measure (1) the feasibility of delivering the adapted intervention and (2) clinic team and patient perspectives on the intervention, in preparation for a future trial on engagement and retention in the PrEP cascade among women.

**Methods:**

We previously applied the ADAPT-ITT (Assessment, Decision, Adaptation, Production, Topical experts, Integration, Training, Testing) model to develop a culturally appropriate, evidence-based intervention, responsive to Black women’s HIV prevention needs. In the present study, we set out to complete the *Training* and *Testing* phases: namely, to hire and train a PrEP navigator and to train clinic staff to deliver the adapted intervention. We completed a 4-month pilot to assess the feasibility of delivering the intervention and collecting outcomes of interest and initial outcome trends (compared to baseline). We further assessed the clinic team and patient perspectives on the intervention to understand the potential for future scale and delivery.

**Results:**

The clinic team participants found the adapted Women’s PrEP project (W-PrEP) intervention both highly feasible and relevant to patients, with minimal impact on clinic flow. Patient participants reported that the W-PrEP intervention was highly relevant and appreciated the education and counseling from the PrEP navigator—many learning about PrEP for the first time from the navigator and health care provider. The outcome measures were both feasible to collect and appropriate to capture the primary outcomes of interest. Finally, the W-PrEP intervention increased the proportion of patients counseled about PrEP from 65% to 76% of patients seen (*P*<.001).

**Conclusions:**

The intervention and outcome data collection was feasible, and open-ended clinic team and patient perspectives showed positive feedback about the intervention and relevance. The associated increase in PrEP counseling is promising but necessitates further evaluation of the effects of the W-PrEP intervention on the PrEP cascade (eg, initiation, persistence, adherence) in a larger randomized trial.

## Introduction

### Background

Cisgender women (subsequently referred to as “women”) account for approximately 6400 preventable new HIV diagnoses in the United States annually, underscoring the unrealized opportunity for HIV prevention programs specifically tailored for women [1]. The HIV epidemic in the United States disproportionately affects women of color and of reproductive age [2-4]. In 2019, despite only accounting for 12% of the population, Black women accounted for 54% of new HIV cases among women; reproductive-age women (25‐44 y) comprised 51% [1]. HIV transmission occurs primarily through receptive vaginal heterosexual transmission (84%) among women [1,5] and is preventable through consistent use of barrier protection and HIV preexposure prophylaxis (PrEP) [6-12]. Barrier protection use, specifically male condoms, is limited by partner power discordance and is dependent upon partner cooperation [13], highlighting the importance of women-controlled HIV prevention options, such as PrEP.

PrEP with daily oral tenofovir disoproxil fumarate/emtricitabine, injectable cabotegravir bimonthly, or injectable lenacapavir biannually reduces HIV transmission by up to 100% in women [7-12]. Additional pharmacoprevention options are in the regulatory pipeline [14,15]. Both daily oral and longer-acting PrEP formulations offer women-controlled options for HIV prevention prior to risk exposure, circumventing the need for cooperation from sex partners. Despite the high efficacy, PrEP is underutilized by women, particularly women of color, in the United States [1,16-19]. The number of prescriptions of PrEP to women in the United States is dwarfed by the number of women with indications for PrEP; there is a considerable unmet need for pharmacoprevention among women [1,16-19]. While the absolute number of women initiating PrEP has increased from 3695 in 2012 to 28,870 in 2021, PrEP uptake among Black women lags far behind other groups [18-20]. Between 2014 and 2017, Black women comprised only 33% of women who initiated PrEP nationally; in contrast, they represented the majority (59%) of new HIV diagnoses among US women in 2017 [17]. Evidence-based interventions are urgently needed to improve PrEP utilization and HIV prevention among women.

### Intervention Development

In response to the lack of evidence-based interventions to improve PrEP initiation, adherence, and persistence among women in the United States, we adapted 2 Centers for Disease Control and Prevention (CDC) Best Practices for HIV Prevention for women (HIV PrEP Services for Urban Women [21,22] and Project Shikamana [21,23,24]) and developed the Women’s PrEP Project *(*W-PrEP), a clinic-based, socio-structural intervention [25]. The W-PrEP intervention was designed to provide education, resources, and support to empower and enable sexual and reproductive health clinics to provide high-quality, nonstigmatizing, universal PrEP services to women who may have increased exposure to HIV, using the ADAPT-ITT (Assessment, Decision, Adaptation, Production, Topical Experts, Integration, Training, Testing) model and guided by the Socioecological Framework [25-27]. We previously published on the formative work for the first 6 ADAPT-ITT steps of this project [25].

In this paper, we describe the last two phases of the ADAPT-ITT process, namely *Training* peer navigators and clinic staff and pilot *Testing* of the feasibility and acceptability of the intervention. The mixed methods pilot data collection reported in both manuscripts was guided by the Practical, Robust, Implementation, and Sustainability Model (PRISM*)* framework, including formative qualitative data collection on context and organizational and patient perspectives [28]. To evaluate the *Training* and pilot *Testing* phases, we focused on the impact of the PRISM context-informed approach on Reach, Effectiveness, Adoption, Implementation, Maintenance (RE-AIM) quantitative outcomes, specifically Reach, Effectiveness, Adoption, and more broadly capturing stakeholder perspectives and context by understanding perceptions of the intervention via qualitative interviews. In the following pages, we describe the pilot implementation and evaluation of the W-PrEP trainings and clinic-based, socio-structural intervention. Using the PRISM framework, we measure the feasibility of delivering the intervention and collecting outcomes of interest and initial outcome trends compared to baseline; we further assess the clinic team and patient perspectives on the intervention to understand the potential for future scale and delivery.

## Methods

### Ethical Considerations

We received approval from the MedStar Health Institutional Review Board (#0006908). Electronic informed consent was obtained prior to all research activities, namely questionnaires and semistructured informational interviews. All study data were deidentified prior to analysis and stored behind a firewall. We compensated participants US $10 for completing brief poststudy visit questionnaires and US $100 for completing 30‐ to 60-minute semistructured interviews.

### Evaluation of the W-PrEP Intervention

In this pilot study, we evaluated the feasibility, acceptability, and initial effects of the multifaceted, clinic-based, socio-structural intervention, which included (1) clinic-wide training and PrEP clinical navigator training, (2) toolkits for clinic roll out and toolkits for each clinical role, (3) increased availability and accessibility of PrEP follow-up options (eg, telehealth, off-site labs), (4) PrEP clinical navigation, and (5) patient-facing educational and support resources. Table 1 summarizes the socio-structural components of the adapted W-PrEP intervention [25]. Clinic-wide trainings and PrEP clinical navigation trainings for the pilot intervention were completed during November and December of 2023. Toolkits and clinic-team facing resources were rolled out in tandem with the trainings. Pilot testing of the intervention ran from January to April 2024, including the assessment of the feasibility of collection of the proposed measures for a planned multicenter, cluster randomized controlled trial. July through October 2023 served as a historical control baseline.

**Table 1.** Adapted W-PrEP^a^ intervention components.

Component	Description
Clinic-based PrEP^b^ rollout toolkit	W-PrEP^b^ implementation checklist Algorithm for universal PrEP counseling and services Implementation algorithm(s) for roll out of new PrEP formulations (including prior authorization) PrEP navigator job description Identification of a PrEP champion
PrEP champion	Medical provider to serve as a PrEP expert and site PrEP champion Responsibilities include facilitation of trainings, consultation on clinic-based implementation of PrEP services, and advising on complex clinical cases
Clinic-wide training	Key training topics include the following: HIV testing (which test at which time) and STI^f^ testing Taking a patient-centered sexual and social history Positive reframing prevention communication/counseling around PrEP How to incorporate patient-centered PrEP and nPEP^c^ provision for women into routine care PrEP initiation, continuation/persistence, and discontinuation nPEP initiation and transition to PrEP Implicit and explicit bias training Trauma-informed care Epidemiology of the US HIV epidemic Social and structural determinants of health related to HIV Key features: Synchronous, interactive PowerPoint presentations scripted for reproducibility and scalability Asynchronous prerecorded modules to accommodate irregular schedules of medical providers and rotating learners, with activities to engage the asynchronous individual learner Centering of the patient voice and experience throughout both modules
Increased availability and accessibility of PrEP follow-up	Increased availability of telehealth/virtual visits for PrEP follow-up visits. These visits are designed as alternatives to in-person visits for women not ready to start PrEP but want to learn more about PrEP (from providers and PrEP navigator) or for women who have been prescribed/started PrEP to follow up regarding issues with prescription pick-up, medication side effects, adherence, or other concerns. Off-site laboratory referrals: providers to offer referrals to off-site commercial laboratories for follow-up STI and HIV testing if more convenient PrEP texting with PrEP navigator: email, text, and telephone follow-up options to address concerns/offer education
Clinic team toolkit	EHR^d^ PrEP order set (eg, STI screening lab orders, PrEP injection or prescription orders, HIV testing orders) EHR PrEP quick-text to facilitate documentation of PrEP education/counseling and prescription or EHR PrEP prompts to remind and encourage providers to counsel all patients about PrEP (dependent upon EHR settings) PrEP/nPEP badge buddy: an identification-badge card sized PrEP/nPEP reference “cheat” sheet with recommended STI and HIV screening, dosing information, and follow-up PrEP/nPEP reference “cheat” sheet and checklists: full-size reference sheet and PrEP start/continuation checklists for quick reference during a busy clinic day (recommended to be posted at the provider work station) Sample scripts for introduction to PrEP (for medical assistants/nurses) and sample scripts for introduction and counseling for prescribing providers “Ask me about PrEP!” swag (eg, pins, lanyards)
PrEP navigator training	In addition to the clinic-wide PrEP training, through a combination of tailored one-on-one training from a PrEP education and navigation expert, independent learning, and online modules, the PrEP navigator training includes the following: PrEP navigation through the “Please PrEP Me” Navigator training [29] Implicit biases in the health care setting [30] Trauma-informed care [31] Introduction to reproductive/sexual health topics (led by the PrEP champion or site health care provider, topically tailored to the clinical context)
PrEP navigator toolkit	In addition to the above clinical tools, the PrEP navigator‘s toolkit includes the following: PrEP quick-text to facilitate documentation of PrEP navigator education/counseling, including assessment of barriers to PrEP Sample script for introduction to PrEP and counseling about PrEP Patient-facing educational resources (see below)
PrEP navigation and support services	Informed by our formative work, PrEP navigators will be cisgender women, ideally from the same community the clinic serves and with first-hand experience with ARVs^e^ through PrEP and/or HIV. PrEP navigators’ job description includes the following services: PrEP educational services: introducing PrEP to patients, combatting misperceptions/myths about PrEP PrEP social and adherence support: breaking down social barriers to PrEP and mitigating the internalization of PrEP-related stigma; building/encouraging individual agency/self-efficacy to initiate and adhere to PrEP PrEP navigation services: facilitating access to PrEP appointments; assisting with insurance authorization; referrals to indicated social services (eg, substance use, gender-based violence, mental health)
Patient-facing PrEP resources	Videos on PrEP and women’s health/sexual health topics (selected by the clinical site) spliced to play on loop in the clinic waiting room to increase awareness of PrEP Inclusive pamphlets on PrEP available in the waiting room and exam rooms Inclusive posters about PrEP and HIV prevention displayed in the waiting room and exam rooms W-PrEP^b^ educational website; QR code distributed by the PrEP navigator and available in the waiting area

a *W-PrEP*: women’s PrEP project.

b PrEP: preexposure prophylaxis.

c nPEP: nonoccupational postexposure prophylaxis.

d EHR: electronic health record.

e ARV: antiretroviral

f STI: sexually transmitted infection

### Study Setting

Pilot testing was conducted in a family planning and preventative care clinic housed in a large tertiary care center in Washington DC. Washington DC is an epicenter of the US HIV epidemic. At the time of the intervention development and pilot study, the prevalence of HIV among women in Washington DC was 1209 per 100,000, nearly 7-fold the national average (170 per 100,000) [32]; the overall prevalence among women was 0.9%, but nearly double (1.7%) among Black women [32]. At the time of this work, Black women represented 1 in 4 new HIV diagnoses in Washington, DC, and 90% of new diagnoses among women [32].

The selected clinic provides over 1300 family planning patient encounters annually, including pregnancy options counseling, pregnancy termination, contraceptive counseling and provision, and sterilization services. The clinic has 2 dedicated physicians, rotating residents, and students; 2 nurses; 2 medical assistants; and 1 dedicated front desk staff member. Since 2017, the clinic has offered PrEP services, initially as a research initiative (with provider and staff trainings completed in 2017) [33], and now as standard of care. We selected this site, given the demonstrated feasibility of studying PrEP interventions in this setting in order to show proof of concept prior to conducting a multisite study [33]. We previously published that 98.4% of women seen in this clinic in 2017 met CDC eligibility criteria for PrEP [33].

### Formative Work

As described previously [25], as part of our formative ADAPT-ITT process, we conducted 10 key informant interviews with community leaders, operations managers, social workers, clinicians, and health educators working in the PrEP field. These stakeholder interviews, in turn, informed a series of focus groups based on *PRISM* domains to gain insight on how best to adapt the two base cases (ie, HIV PrEP Services for Urban Women [21,22] and Project Shikamana [21,23,24]). We conducted focus groups with providers, PrEP educators and navigators, and patients. With the information gathered, we adapted the base cases into preliminary prototypes for the W-PrEP training and content delivery; these prototypes then underwent theater testing and evaluation by topical experts prior to finalization of the W-PrEP model to be pilot tested [25].

### Pilot Implementation Testing Study Design

To assess the implementation of the W-PrEP intervention in the pilot *Testing* phase, we utilized a quasi-experimental, interrupted-time series (ITS) design, specifically a 4-month baseline period followed by 4 months of intervention delivery. An ITS design was chosen to allow internal comparison of the intervention’s reach and effectiveness to the historical baseline period. ITS was additionally chosen for this pilot to assess the suitability of the design to assess within-site changes in the proportion of patients engaged/retained in the PrEP cascade associated with the intervention to be utilized in a subsequent cluster randomized controlled trial.

### Outcome Assessment

In preparation for a future multicenter, cluster randomized controlled trial, outcomes were guided by the select elements of the PRISM framework [28], namely Reach, Effectiveness, Adoption, and Organizational and Patient Perspectives on the intervention (Table 2). Still, at this stage, we focused on the feasibility of measuring the quantitative outcomes of interest. We defined *Reach* as the proportions of eligible patients who received PrEP counseling by the PrEP navigator or a medical provider as documented in the electronic health record (EHR) or navigator log; eligibility was defined by the 2021 CDC criteria [34]. We defined *Effectiveness* as the proportion of eligible patients who initiated PrEP and as the measured change in perceived medical bias on the postvisit questionnaire. We collected this data primarily to assess the feasibility of the measures, as well as to conduct descriptive statistics and to calculate standard deviation to inform planning for the subsequent full-scale trial. We defined *Adoption* as the proportion of clinic team members who participated in the clinic-wide training and by the relative change in knowledge of PrEP and PrEP best practices on the pre- and posttraining evaluation. At this stage of the work, we did not quantitatively measure implementation fidelity, as we planned to have the pilot be pragmatic, allowing for further adaptation of W-PrEP as needed. From PRISM, we assessed the *organizational perspective* through interviews with the clinic team and PrEP navigators to evaluate perceived acceptability and feasibility of implementing the intervention and satisfaction with the training and the intervention. We also assessed the *patient perspective* via in-depth interviews focusing on the educational resources and counseling provided by the PrEP navigator and by medical providers.

**Table 2.** Evaluation of the pilot using the PRISM framework.

PRISM component	Measure
Reach	Percentage of eligible patients who received introduction to PrEP^a^ by navigators (by comparison of the navigators counseling log to the EHR^c^) Percentage of eligible patients counseled about PrEP by providers (by EHR review)
Preliminary effectiveness^b^	Percentage of eligible patients who initiate PrEP (by EHR) Percentage of change in perceived medical bias (by online postvisit survey)
Adoption	Percentage of clinic-team members who participated in training (by attendance log) Percentage of change in PrEP knowledge and PrEP best practices (pre-/postclinic-wide training questionnaire)
Intervention characteristics*:* organizational and patient perspectives	Perceived feasibility of implementing the intervention and identified barriers (by IDIs^d^ with the clinic team and navigators) Satisfaction with training and intervention usability/complexity among clinic team and navigators (by IDIs with the clinic team and navigators) Acceptability of the provider or navigator PrEP education/counseling, focusing on burden and addressing patient barriers (by IDI with patients)

a PrEP: preexposure prophylaxis.

b For the feasibility of data collection and calculation of standard deviation, *not* for statistical testing in a pilot.

c EHR: electronic health record.

d IDI: in-depth interview.

### Quantitative Tools and Outcome Data Collection

For patient-level characteristics and outcomes, the research team extracted patients’ demographics, indications for PrEP by the 2021 CDC eligibility criteria, documented counseling about PrEP by PrEP clinical navigators and by providers, level of interest in PrEP (if recorded), and follow-up for subsequent PrEP visits (eg, for PrEP education, PrEP initiation, and PrEP continuation at 1 mo) from EHR. Additionally, in the baseline and intervention periods, we invited all patients to complete a 5-minute, smartphone-based, post-visit questionnaire, which queried sociodemographics, indications for PrEP, perceived medical discrimination [35], norms around and attitudes toward PrEP, intention to initiate PrEP, and interaction with the W-PrEP intervention. Given that many patients participating in routine care were offered analgesia and sedation for medical procedures, the postvisit questionnaire was offered via a flyer distributed by the front desk with a QR code for questionnaire completion at home and via email within a week of their appointment. Quantitative EHR and questionnaire data were entered into REDCap (Research Electronic Data Capture [36,37]) hosted at MedStar Health. Additionally, the PrEP clinical navigators kept a log of all patients seen, education provided, and planned follow-up as applicable. For clinic-level outcomes, we utilized attendance logs to capture attendance at the clinic-wide and PrEP clinical navigation trainings. At these trainings, participants completed a pre- and posttraining assessment of PrEP knowledge and understanding of best practices, as well as a postassessment evaluation of the training.

### Quantitative Analysis

We calculated descriptive statistics, including frequencies and percentages, measures of central tendency, dispersion, and appropriate visualization approaches (eg, box plots and spaghetti plots) on each outcome variable; we used line graphs to illustrate the trends of the outcome over time. Bivariate analyses were conducted, including *t* tests for normally distributed continuous variables and *χ*^2^ tests or Fisher Exact tests for categorical variables, as appropriate based on cell sizes, to compare sample characteristics between the intervention and historical-control time periods; nonparametric methods were used in the presence of nonnormally distributed variables. We conducted multilevel modeling (MLM) with implementation of intervention (post vs pre) to gage a preliminary assessment of the intervention effect, accounting for possible repeated measures within some participants over the period. Specifically, MLM assessed each outcome variable in a separate model, including the predictor as intervention status (post vs pre), with a random intercept at the patient level to account for the possible multiple visits (44% with 2 or more visits) within each patient, before and after adjusting for sample characteristics (eg, age, race, ethnicity). We conducted ITS analyses, including an interaction between time in month as a continuous variable, which is centered at the first month after intervention implementation, and a categorical variable indicating intervention (post vs pre) in MLM. A significant coefficient for intervention in the model indicates an immediate change after intervention, and a significant interaction indicates a change in the trend over time between pre- and postintervention periods. Of the 1437 patient encounters in the dataset, 1 (0.1%) encounter was missing the month of the visit and the intervention status, 2 (0.1%) were missing age, 12 (0.8%) were missing race, and 27 (1.9%) were missing ethnicity. Eight were missing confirmation of the starting of PrEP, while 1 encounter was missing other outcomes. A higher percentage of missingness was found for the other race categories, 12.2% versus a range of 1% to 3% for Black, White, Asian or Pacific Islander, or Native American (*P*<.001). Those with any missingness or those with complete data did not differ in age, ethnicity, or any outcome. Listwise deletion was applied for the covariates, including age, race, and ethnicity, while maximum likelihood was used for the outcome if it was missing in MLM. A significance level was set at 0.05 for all the analyses.

### Qualitative Tools and Data Collection

At the end of the *Testing* phase, we conducted semistructured in-depth interviews to assess *perspectives on the intervention* with clinic staff, PrEP navigators, and patients who participated in the pilot. See Multimedia Appendix 1 for interview guides. All clinic team members and PrEP navigators were invited to participate in interviews; interview questions focused on perceptions of the intervention, including trainings and the PrEP clinical navigator program, and potential improvements to be incorporated. We utilized purposive sampling to recruit a total of 20 patient-participants, specifically Black women of reproductive age, at varying stages in the PrEP cascade (ie, not interested in PrEP, considering initiating PrEP, initiated PrEP). These patient interview questions focused on the patient experience with the intervention and potential improvements to the intervention and provision of PrEP services. Interviews were conducted by experienced research scientist team members. Given the timing during the COVID-19 pandemic and to increase access for participants who would be unable to attend in person, all interviews were conducted remotely via a secure Microsoft Teams platform.

### Qualitative Analysis

We applied rapid qualitative analysis (RQA) [38-40] to systematically translate the qualitative data from the interviews into actionable refinements. We audio-recorded interviews with the consent of participants, which were then transcribed verbatim. We developed summary templates to identify the coder, respondent role, and coding domain (ie, strengths, information gaps, actionable improvements). We conducted analyses of a subset of interviews (ie, one from each respondent type) in order to ensure adequate fit of the template to the data, then we coded the remaining transcripts. For the *Training* and *Testing* ADAPT-ITT phases, we developed and populated matrices to align with germane components of the PRISM implementation framework. We used the matrices to synthesize key themes during intervention development and to synthesize strengths, information gaps, and other actionable improvements (eg, unclear or confusing language, alternative or additional resources, and toolkit components), stratified by respondent type or role. These summaries guided team discussions and refinement decisions pertinent to the W-PrEP intervention. All RQA analyses were performed by two trained medical student research team members with experience in qualitative analyses. RQA were completed under the supervision of the study’s primary investigator, the study implementation scientist, and the study health communications consultant.

## Results

### Training

#### Clinic-Wide Training

The adapted training was divided into two modules, each approximately 1 hour long. The first module focused on HIV prevention, offering a brief introduction to HIV and HIV transmission, HIV tests and timing of HIV testing, and HIV prevention, with a focus on PrEP and nonoccupational postexposure prophylaxis (nPEP). The training detailed the PrEP modalities and regimens approved for protection against vaginal exposure to HIV, indications for PrEP and nPEP, and the “PrEP Steps”: taking a sexual and social history, education and counseling, laboratory testing, and management of starting, continuing, and stopping PrEP. The PrEP steps were interspersed with patient testimonials, group activities (eg, writing a script to introduce PrEP to all patients with nonstigmatizing language), an original video created by the research team modeling trauma-informed, PrEP-positive care, and sample cases for group discussion. The first module also focused on the incorporation of PrEP into clinical practice.

The second training module focused on the epidemiology of the HIV epidemic in the United States—specifically for women. The training reviewed the social and structural determinants of health as related to HIV and HIV prevention for women. It discussed trauma-informed care and incorporation of trauma-informed care into HIV prevention care. The second module also reviewed strategies to mitigate bias in clinical practice. Both training modules incorporated patient testimonials, group activities, and a component of the original video highlighting social and structural determinants of health barriers to care.

#### Adoption

The clinic team elected to complete both modules in single training days. Two training days were scheduled to accommodate training of clinic team members, including the PrEP navigators. The training was open to other medical providers within the department; all members of the clinic team, including the 2 PrEP navigators, completed the training. Clinic team members were additionally joined by several resident trainees and one certified nurse midwife who had expressed interest in completing the training. A total of 13 individuals completed the trainings, along with deidentified, linked pre- and posttests for each module. Attendance was monitored via sign-in sheet or attendance log. Combined knowledge test scores improved from a mean of 65% to 89% between the pre- and posttests. Participants favorably reviewed the training in the feedback section of the posttest, and 100% of participants reported “intend(ing) to apply what (they) learned in the training” and feeling “motivated to make changes in (their) work” Table 3 highlights exemplary quotations regarding the trainings from the feedback portion of the posttest.

**Table 3.** Exemplary quotations from the qualitative evaluation of the training and pilot testing.

Interview topic, theme	Quotations
Training	
Most useful component of training: content on guidelines	“(Review of) guidelines for prescribing and monitoring” -Training participant #11
Most useful component of training: content on patient-centered care	“Hearing patient voices (and) seeing examples of “bad” and “good” clinical scenarios. While we roll our eyes over shame tactics, mistreatment (by health care providers) it’s always helpful to see it so clearly and remind us to be better” -Training participant #4
Main take-away: content on guidelines and universal approach to PrEP^a^ education	“I can prescribe PrEP and should be talking to pretty much all of my patients about it” - Training participant #1
Main take-away: content on stigma	“(To) encourag(e) the topic of PrEP to become normal and less stigmatized in clinic settings” - Training participant #8
Most useful component of training: format of interactive exercise	“The elevator pitch and counseling for PrEP” (referring to the toolkit scripts and interactive exercise) - Training participant #10
Most useful component of training: content on clinical scenarios	“The training made sure to…go through…scenarios that we might encounter. Some of those scenarios did end up happening, so I felt like I was better equipped to handle certain conversations because (of) that training” - Training participant #2
Testing	
Perception of the intervention: PrEP navigation	“It’s very helpful in terms of giving more information to our patients about PrEP, so that we’re not…starting at Ground Zero to discuss PrEP, ‘cause obviously …there’s all these time crunches and … it’s…one more thing to crunch into the time. And so while I review it with every patient, if I’m not starting from scratch, when I’m reviewing it, then it’s a big help.” - Clinic team member #2: medical provider, White cisgender female
Perception of the intervention: PrEP educational resources	It serves a very important purpose because…most of our patients are high risk for HIV and (they) hear about PrEP (in the educational videos) on (the) TV and actually having someone to talk to them more in depth…(and) we give them pamphlets. Some of them may say no initially, but when they go home they read the pamphlet.” - Clinic team member # 4: Nurse, Black, cisgender female
Perception of the intervention: barriers to PrEP	“With the patients that I encountered a lot of them didn’t know that (PrEP) was also…for women. A lot of them just assumed it was for males, gay males, …and they also appreciated the fact that this was something that was being provided to the women, especially the minorities in our community. …I think also being able to have the conversation with them maybe helped with the stigma part of it.…(A) lot of them became more comfortable in talking about HIV and HIV prevention because initially when I was talking to certain patients about it…they seemed like they were hesitant about talking about it, but the more I went into detail…they were …a bit more comfortable having the conversation.” - PrEP Navigator #1: Black, cisgender female
Perception of the intervention: PrEP navigator	*“*She gave me an overview of what exactly PrEP is, how I can go about getting it, and if I’m interested, the steps that I could take to link up with her and move forward...I became informed of what PrEP was. …She also gave me the option of starting then or setting a date for myself, or even if I wanted to take a deeper dive and learn more. She provided those resources, and I was grateful for that…that was a generous gesture. I feel like being knowledgeable and aware of what’s happening around you, especially for young women or men engaging in sexual activity, is important. Preventative care and hygienic care are essential. I appreciated having someone there to be informative, even if I couldn’t proceed at that moment. It was very informative, and now I can assist others and have conversations.” - Patient #18: Black, cisgender female, age 23, considering PrEP
Perception of the intervention: PrEP navigator	"My experience was great. Even though I might not remember all the details, the information was presented well. I’d like to have (the PrEP navigator) on my team to help me stay on track with PrEP. It’s a process with appointments and doses, so I could use that reminder.” - Patient #15: Black, cisgender female, 31, considering PrEP

a PrEP: preexposure prophylaxis.

#### PrEP Navigator Training

The PrEP clinical navigator training program consisted of the above clinic-wide training and a curated curriculum centered on the Please PrEP Me navigation manual [29]. PrEP navigators independently studied the Please PrEP Me navigation manual and then reviewed it with the PrEP navigation team from a local health center over two sessions, including talking through potential challenging scenarios and role plays. Additionally, the PrEP navigators independently completed online trainings on trauma-informed care [31] and implicit bias in the clinical setting [30], which were subsequently discussed as a small group. Lastly, navigators also completed tailored training on sexual and reproductive health, reviewing commonly used anatomical and medical terms in reproductive and sexual health care, and common conditions and medical indications for visits in the pilot clinic setting. The training was well received; the review of clinical scenarios and role plays was highlighted as particularly helpful. Table 3 highlights representative quotations regarding the trainings.

### Testing

#### Instrumentation

We found the instrumentation and outcome measures to be both feasible to collect and appropriate to capture the primary outcomes of interest in the EHR. However, during the preimplementation period, only 54 respondents completed the survey due to competing priorities or sedation during the appointment, making it inappropriate and not feasible to continue survey data collection in clinic.

Interviews among clinic staff (n=5) and navigators (n=2) in the last weeks of the implementation period showed general perceptions of feasibility and acceptability of the training and intervention delivery, noting that the intervention minimally impacted clinic workflow.

#### Reach and Preliminary Effectiveness

**Figure 1.**
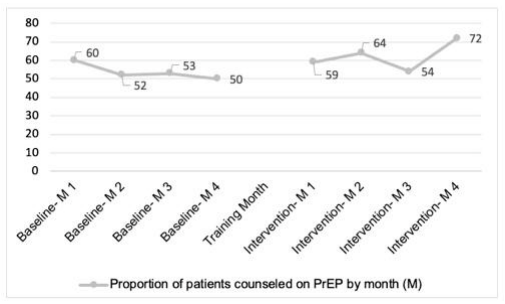
Proportion of patient visits with documented preexposure prophylaxis (PrEP) counsel by a health care provider or by a PrEP navigator by month (M) during the baseline and intervention periods (not controlling for multiple follow-up visits).

We assessed the frequency of PrEP counseling by providers in the preimplementation period (July to October 2023) and compared the proportion of patients who received counseling on PrEP. We found that provider counseling was maintained for 6 years after the previous PrEP training, with counseling documented in 65% (350 of 537) of all patients seen during the 4-month period (controlling for patients with multiple follow-up visits). In the setting of already high provision of PrEP counseling, PrEP counseling *by providers* did not significantly increase during the intervention period; however, with the introduction of the PrEP clinical navigators, the reach of the W-PrEP intervention increased counseling to 76% of patients seen (controlling for patients with multiple follow-up visits). Based on MLM, the likelihood of PrEP counseling increased by 43% in posttest without covariates (odds ratio [OR]=1.43, 95% CI 1.16‐1.77; *P*<.001) and after adjusting for covariates (adjusted OR [aOR]=1.51, 95% CI 1.21‐1.88; *P*<.001). The ITS showed that there was a significant immediate change in the odds at the first month after the initiation of intervention implementation (aOR=1.82, 95% CI 1.02‐3.24; *P*=.04), and there was also a significant increase in the trend in PrEP counseling (aOR=1.28, 95% CI 1.05‐1.57; *P*=.02). We successfully collected data on preliminary *Effectiveness,* namely PrEP initiation, to demonstrate feasibility of data collection and calculation of standard deviation, *not* for statistical testing in a pilot. We found a statistically significant increase in interest in learning more about PrEP (n=39, 5.6% → n=93, 12.5%; *P*<.001); we did not see statistically significant increases in prescription of PrEP/initiation of a prior authorization nor differences in PrEP initiation but were not powered to do so. Table 4 describes the demographic characteristics, indications for PrEP, and PrEP-related outcome measures collected in the pilot. Figure 1 depicts the reach of counseling by providers or navigators by month in the baseline and intervention periods.

**Table 4.** Demographic characteristics, indications for PrEP^a^, and PrEP-cascade outcomes by patient encounter during baseline and intervention periods of the pilot study^b^.

Demographic characteristics, indications for PrEP, and PrEP-cascade outcomes	Baseline	Intervention	*P* value
Number of participants, n (%)	691 (48.1)	745 (51.9)	
Age (y), mean (SD)	29 (7.4)	30 (7.0)	.34
Race, n (%)			.06
Black	501 (73.8)	546 (73.3)	
White	102 (15)	85 (11.4)	
Asian/Pacific Islander	13 (1.9)	18 (2.4)	
Native American	2 (0.3)	1 (0.1)	
Other (including mixed race)	61 (9)	95 (12.8)	
Ethnicity, n (%)			.35
Hispanic	36 (5.3)	48 (6.5)	
Non-Hispanic	637 (94.7)	688 (93.5)	
Inconsistent condom use, n (%)			.02
No	348 (50.4)	420 (56.4)	
Yes	343 (49.6)	325 (43.6)	
STI^h^ diagnosis within the past 6 months, n (%)			.46
No	683 (98.8)	733 (98.4)	
Yes	8 (1.2)	12 (1.6)	
IV drug use or needle sharing, n (%)			
No	691 (100)	745 (100)	
Partner(s) status^c^, n (%)			<.001
Unknown HIV status	0 (0)	175 (23.5)	
Not documented	690 (100)	567 (76.2)	
Positive	0 (0)	2 (0.3)	
Requests PrEP, n (%)			.59
No	688 (99.6)	743 (99.7)	
Yes	3 (0.4)	2 (0.3)	
nPEP^d^ use, n (%)			
No	691 (100)	745 (100)	
PrEP navigator introduced PrEP/counseled patient regarding PrEP, n (%)			<.001
No	N/A^e^	462 (62)	
Yes	N/A	283 (38)	
Provider introduced PrEP/counseled patient regarding PrEP, n (%)			.02
No	323 (46.8)	395 (53.2)	
Yes	367 (53.2)	347 (46.8)	
Either provider or navigator introduced PrEP/counseled patient regarding PrEP, n (%)			<.001
No	324 (46.9)	284 (38.1)	
Yes	367 (53.1)	461 (61.9)	
Patient expressed interest in learning more about PrEP, n (%)			<.001
No	652 (94.4)	651 (87.5)	
Yes	39 (5.6)	93 (12.5)	
Patient expressed interest in starting PrEP, n (%)			.32
No	680 (98.6)	729 (97.9)	
Yes	10 (1.4)	16 (2.1)	
Provider prescribed TDF/FTC^f^ or initiated prior authorization for CAB^g^, n (%)			.48
No	686 (99.3)	736 (98.9)	
Yes	5 (0.7)	8 (1.1)	
Verified PrEP initiation, n (%)			.66
No	685 (99.3)	734 (99.5)	
Yes	5 (0.7)	4 (0.5)	

a PrEP: preexposure prophylaxis.

b Fisher exact tests were conducted if any cell size is under 10.

c Partner HIV status was added to the EHR “quick text” for the W-PrEP Intervention.

d nPEP: nonoccupational postexposure prophylaxis.

e Role of PrEP navigator did not exist prior to W-PrEP Intervention.

f TDF/FTC: tenofovir disoproxil fumarate/emtricitabine.

g CAB: cabotegravir.

h STI: sexually transmitted infection

### Organizational and Patient Perspectives on the Intervention

Multiple interviewees noted patient interest in PrEP education but challenges with continued low patient-perceived risk of HIV and subsequent low PrEP uptake. Multiple members of the clinic team mentioned that due to the time lag associated with the prior authorization for long-acting cabotegravir, it was challenging to comply with the CDC guidelines which recommend negative HIV testing within 7 days prior to initiation [34]. The clinic team universally praised having a PrEP clinical navigator to expand patient education, assist with prior authorizations, and orchestrate appointments for long-acting PrEP. They additionally lauded the expanded training, enhanced toolkit, and patient-facing resources. Patient-participants (n=20) interviewed likewise reported that their exposure to the W-PrEP intervention was highly acceptable and appreciated the education and counseling from the PrEP navigator—many learning about PrEP for the first time from the navigator and provider. A common sentiment was that although the information about HIV and PrEP was insightful, it did not apply to them. Table 3 highlights exemplary quotations regarding the W-PrEP intervention and implementation.

## Discussion

### Principal Results

The adapted W-PrEP intervention was responsive to the concerns and expressed needs of health care providers and clinic team members, PrEP navigators, and women of reproductive age, in particular Black women. There was high adoption (100%) of the training by clinic team members and significant improvement in clinic team knowledge of HIV prevention and PrEP. Though this pilot was not powered to assess increased PrEP uptake, W-PrEP demonstrated increased reach of PrEP counseling with the addition of the PrEP clinical navigator, dramatically increased patient interest in learning more about PrEP, and a nonsignificant trend toward increased prescription of oral PrEP/prior authorization request for injectable PrEP on the part of providers. Of note, we did observe a small decrease in provider-led PrEP counseling from baseline. In discussion with the clinic providers to understand this decrease, providers mentioned that if the PrEP navigator counseled a patient about PrEP and signaled a patient’s disinterest in discussing PrEP further, the providers were then less likely to rebroach the topic in that visit. Clinic team members, PrEP navigators, and patients found W-PrEP highly acceptable. Clinic team members found the training and implementation of the intervention feasible, with minimal interruption of clinical flow. In terms of study instrumentation, we found that EHR review was highly feasible to assess our primary outcomes. In the context of the family planning clinic, the self-report survey tool was not feasible due to low response; in clinical settings without conscious sedation for procedures, this measure would have been more feasible as patients could complete the questionnaire on-site immediately postvisit.

### Comparison to Prior and Contemporary Work

Despite increased public health marketing directed to women and direct-to-consumer advertising by industry targeting women, there remain limited CDC-designated best practices for HIV prevention or PrEP uptake inclusive of women, let alone focusing on women [41]. Coleman et al [42] describe a clinic-based integrated pharmacy and PrEP navigation program, inclusive of patients of all sexes/genders, designed to break down barriers to PrEP utilization. Cisgender women comprised a tiny minority of the patients included, and, although not statistically significant, both the proportion and absolute number of cisgender women decreased from the baseline to the intervention period. Casey et al [43] describe a successful educational intervention across primary care and women’s health clinics within the New York City public health care system, with significant increases in the numbers of primary care and women’s health providers offering and prescribing PrEP, similar to the intervention that W-PrEP was adapted from [22]. The scope of the intervention was limited to educating and empowering medical providers to provide PrEP services and did not address the significant social and structural barriers to PrEP uptake and persistence.

W-PrEP expanded upon our solely educational intervention [22,33] to more fully address identified patient-, provider-, and clinic-level socio-structural barriers to PrEP and HIV prevention for women of reproductive age and, in particular, Black women. Specifically, W-PrEP was designed to combat low awareness and stigma about PrEP among patients, low knowledge/confidence of how to provide PrEP among providers, and clinic-level challenges of systems/logistics for obtaining prior authorization and tracking patient follow-up. This strategy demonstrated a significantly increased reach of PrEP counseling and education, resulting in increased interest in learning more about PrEP. Developed in parallel to W-PrEP and currently under evaluation, the POWER Up intervention similarly utilizes a clinic-based multifaceted approach consisting of routine PrEP education for patients, standardized provider training, EHR optimization, PrEP navigation, and PrEP clinical champions [44]. Both interventions appear to share a universal approach to patient PrEP education, a PrEP navigation program, utilization of PrEP clinical champions, and EHR toolkits. W-PrEP differs from POWER Up in several aspects: (1) W-PrEP’s clinic-wide training program is inclusive not only of providers but of all clinic staff members, recognizing that patients often receive health information and messaging from both clinical and nonclinical members of the clinic staff. This feature leverages the potential of all members of the team, from the clinic receptionist to the nurse administrator, to provide PrEP resources, field basic questions about PrEP and HIV prevention, and direct patients to providers or PrEP navigators for additional information, as needed. (2) W-PrEP offers several toolkits with practical resources constructed to aid in provision of PrEP education and services. These include a clinic-level toolkit, including modifiable algorithms for incorporating PrEP into clinic flow and a clinic PrEP roll-out readiness checklist, a clinic team toolkit with quick reference “cheat sheets” and badge buddies and sample scripts for introducing PrEP and PrEP counseling, and a PrEP navigator toolkit with scripts and patient-facing educational resources. These toolkits were created in response to providers and administrators' plea for blueprints to integrate PrEP services into busy clinical practice. (3) Finally, W-PrEP offers expanded access through telehealth and web-based follow-up and off-site laboratory referrals.

### Limitations

As this was a pilot, we limited both the number of sites and duration—thus we were not powered for statistical testing of the outcomes planned for a future trial of the intervention. This intervention was piloted in an urban hospital center affiliated with an academic institution; however, we posit that our findings are likely generalizable to sexual and reproductive health centers in both community and academic settings. Future work should incorporate additional geographic regions and clinical settings of varied sizes. It is also worth noting that the pilot was conducted in a setting where providers previously received informational training on PrEP in 2017 and continue to offer PrEP services to the majority of patients. We anticipate that in settings with lower baseline PrEP counseling, the W-PrEP intervention would have a more dramatic effect upon the outcome measures of interest.

Although we were successful in testing the use of EHR data collection for our primary outcomes, we were largely unsuccessful with the implementation of the patient questionnaire. We were unable to utilize the questionnaire immediately postvisit, as a significant proportion of patients received sedation for termination of pregnancy. We anticipate that in nonprocedural clinical settings (or settings that do not routinely utilize sedation), exit questionnaires could be administered by a coordinator after the clinical visit with a higher response rate. We are additionally exploring other data capture systems to simplify the process.

### Conclusions

W-PrEP, a clinic-based, socio-structural intervention adapted from two CDC HIV Prevention Best Practices models, demonstrated increased reach of PrEP counseling, increased interest in PrEP among patients, and a nonsignificant trend toward increased prescription of oral PrEP/prior authorization request for injectable PrEP by providers. The intervention was both acceptable and feasible to the clinic team and the intended patient population. Further evaluation is needed to test the effects of the intervention on the PrEP cascade, namely PrEP initiation, adherence, and persistence, particularly among the intended population.

## Supplementary material

10.2196/80653 Multimedia Appendix 1 Interview guides.

## Abreviations:

ADAPT-ITT: Assessment, Decision, Adaptation, Production, Topical Experts, Integration, Training, Testing
aOR: adjusted odds ratio
CDC: Centers for Disease Control and Prevention
EHR: electronic health record
ITS: interrupted-time series
MLM: multilevel modeling
OR: odds ratio
PrEP: pre-exposure prophylaxis
PRISM: Practical, Robust, Implementation, and Sustainability Model
RE-AIM: Reach, Effectiveness, Adoption, Implementation, Maintenance
RQA: rapid qualitative analysis
SSDOH: Social and structural determinants of health
W-PrEP: Women’s PrEP Project
