# Supplementary material for: “The Women’s PrEP Project,” a Clinic-Based, Socio-Structural Intervention to Improve the Provision of Preexposure Prophylaxis for Cisgender Women: Interrupted Time Series Pilot
Source: JMIR Form Res. 2025 Dec 3;9:e80653. doi: 10.2196/80653 (PMC12674590; doi:10.2196/80653)
Supplement: Multimedia Appendix 1 [file formative-v9-e80653-s001.docx]

**APPENDIX 1.**

**Interview Guide: Patients**

*Thank you for speaking with me today. We have been working on a project to deliver PrEP to women in DC over the last couple of years. As a reminder, or in case you’re not familiar, PrEP is a daily pill or bi-monthly injectable medicine that prevents HIV in case of exposure to the virus. We are interested in speaking with you today to learn about your thoughts on PrEP, experiences with this clinic or clinic staff, and to hear any suggestions you may have for improving and expanding services for women in our community. I hope you’ll feel comfortable speaking your mind. There are no right or wrong answers. We really want to know your opinions and ideas. I assure you that everything we discuss will remain confidential and your opinions won’t affect any services you receive*

1. **Experience with** the PrEP project

*I would like to start by asking you about your experiences with PrEP in the context of your clinical visits and/or interactions with peer navigation.*

1. What are the different ways that you have learned about PrEP?
   1. Can you tell me about any PrEP information you saw in the waiting room?
   2. Can you tell me about the first conversations you had with clinic staff or providers about PrEP?
      1. Which types of providers talked to you about PrEP?
      2. What were the conversations like when staff or providers talked with you about PrEP?
   3. What have you learned about PrEP?
   4. What was most useful to you in helping you better understand PrEP?
2. OK, now I’m going to ask you about the information you received at different times during your visit. Think back to when you received information in the waiting room.
   1. What was most helpful about the information you received in the waiting room?
   2. What do you think should have been included in information provided in the waiting room that wasn’t?
   3. How did you feel about receiving this information on PrEP in the waiting room?
3. Thank you – now I want to ask about the information you received from the clinic staff.
   1. What was most helpful about the information you received from clinic staff?
   2. What do you wish had been included in information provided by clinic staff that wasn’t?
   3. How did you feel about receiving this information on PrEP from the clinic staff?
4. OK, now I’d like to shift gears a little to talk about any conversations you may have had with your provider in the clinic about PrEP. Can you tell me about that?
   1. How did it go?
   2. What went well?
   3. What didn’t you like about talking about PrEP with your provider in clinic?
5. What was your reaction to the information or conversations about PrEP – did they change your thoughts on whether or not it might be a good fit for you?
6. How do you think other women in your community would feel about learning about PrEP in this way?
   1. Is there anything that we should have done differently that we haven’t already discussed?

*If engaged with peer navigation:*

1. In addition to the medical staff at the clinic, did you work with with a “peer navigator” (define)?
2. Tell me about your experience with the peer navigator.

a. What was most helpful about working with the peer navigator?b. What did not go so well when you were working with the peer navigator?

- 1. Was there anything that made it harder for you to work with a peer navigator?
  2. How could peer navigators better support you in learning about PrEP or using PrEP?

1. How does talking about PrEP support your goals in staying healthy?
2. **The PrEP project going forward**

*In the final portion of the interview, I’d like to ask your suggestions and ideas for how we can improve and expand on the activities to expand PrEP services for women going forward.*

1. What do you think other women in your community think about PrEP?

- 1. Do the women you know know of PrEP?
  2. Do you think that this strategy we’re using of talking to women in the clinic would be effective in getting the word out about PrEP?

1. Our goal in having women talk with peer navigators is to help get people going on PrEP if they want to use it. How should peer navigators support women in terms of getting on PrEP?
   1. What would help support women to stay on PrEP if they start?
   2. How could peer navigators support women to help them stay on PrEP?
2. Would you be interested in activities that bring women together in small groups to talk about health topics, including but not limited to HIV prevention?
   1. Would you be interested in that in addition to the 1:1 peer navigators, as an add-on?
   2. What would those groups look like ideally from your perspective?
      1. How big or small should they be?
      2. Where should they meet?
      3. What topics should they cover? Would you like those to be in person or virtual?
3. How do you think your health care team could do better at getting the word out to women about PrEP and helping them consider whether it might be a good fit?
   1. What other activities or services might help women learn more about PrEP and/or decide about PrEP?
4. Is there anything more you would like to share with me about what else you hope the PrEP project could do for women in our community?

*Thank you for your time today. We appreciate your ideas and feedback on the implementation of the project.*

**APPENDIX 2.**

**Interview Guide: Clinic Team (e.g., Providers, Nurses, MAs, Front Desk Staff)**

*Thank you for speaking with me today. As you know we have been implementing a research project to deliver PrEP to cisgender women in DC over the last couple of months. We are interested in speaking with you today to learn about your experiences of how the implementation process of this project has been going and any suggestions you have for its improvement and expansion. There are no right or wrong answers. Please feel free to be as candid as possible. We really want to know your opinions and ideas. Please remember that everything we discuss will remain confidential and nothing you say will be connected to your identity.*

We consider the major components of the project to be:

1. Peer navigation
2. Provider training on considerations around PrEP delivery
3. **Perceptions of the W-PrEP project**

*I would now like to ask you about your perceptions of and experiences with the PrEP project.*

1. What has been your experience to date with the PrEP project?
   1. In what ways have you been involved with the PrEP project?
   2. Can you tell me about your experiences working with the PrEP project? From your point-of-view, how did it go?
2. What purpose do you think the PrEP project serves in terms of the services it provides to clinicians and clinic staff?
   1. How would you evaluate the project in terms of that purpose?
   2. what has worked well from your perspective? Tell me about successes you have seen.
   3. What has worked less well?
      1. What have been the specific barriers you have observed?
3. What shifts have you seen in the relationships between clinical site staff and patients as a result of this project?
4. What kind of changes did you observe in patient knowledge of PrEP during the study period?
   1. What resources were commonly mentioned for learning about PrEP?
      1. Probes: waiting room videos, pamphlets, navigation? Discussion with medical assistant or nurse?

PrEP training (confirm that they attended)

1. Tell me about your experience attending the clinic-wide training with the PrEP project …
   - 1. What was helpful about it? Even though those aspects were helpful, could it be improved in relation to those strengths?
   1. What was less helpful to you?
      1. How could the program be improved in relation to those challenges?
   2. How do you think it changed the way you think, work or perform your duties at the clinic?
   3. What would you change or do differently moving forward?
2. What did you think of the tools provided such as the lanyards or pins saying “ask me about PrEP” or the cheat sheets/quick reference guide, EHR quick text, or sample scripts?
   1. Do you have any favorite tools?
   2. Are there any tools that were not helpful?
      1. Why weren’t they helpful? How could they be improved?

Peer support component:

1. What was your experience with the peer navigation component?
2. What aspects of peer navigation were most helpful to you? Why?
   1. Probes: talking to patients to provide education about PrEP; navigating with insurance; telehealth follow up with patient
3. What aspect of peer navigation was most helpful to your patients?
4. What aspects of peer navigation were least helpful to you? Why?
   1. Probes: talking to patients to provide education about PrEP; navigating with insurance; telehealth follow up with patient
5. What aspect of peer navigation was least helpful to your patients?
6. What worked well with the workflow to provide a warm handoff to the peer navigators?
7. What could be improved in the workflow to provide a warm handoff to peer navigators?
8. **The W-PrEP project** **going forward**
9. What do you think we could do better to improve the impact of PrEP project?

What should we consider as we scale to additional clinical sites and staff?

1. What are we missing?
   1. Are there certain topics the PrEP project could provide trainings on that would be helpful for clinical personnel to know about? Health related topics? Safety?
2. What other thoughts or ideas do you have on how the PrEP project might be able to help support the cis-gender women in this community to be healthy and safe?
3. **Perceptions and views on PrEP**

*Now I would like to talk to you about your perceptions and views on PrEP.*

1. What level of awareness do you think the cisgender women at your clinic have of PrEP?
   1. How aware of it are they? What do they know about PrEP?
   2. How has this changed over the course of the study
2. Thinking about the cisgender women you see at the clinic, what are some of the reasons they decline to use PrEP?
   1. Specific reasons (stigma, costs, concerns about side effects)?
   2. Can you think of any patients who could have really benefited from PrEP, but declined to use it?
      1. Can you tell me about their reactions? Why didn’t they use it?
3. Thinking about the women you see at the clinic, what concerns do you have about adherence?
   1. What factors might prevent optimal PrEP adherence in women?
   2. What specific barriers might they face? (stigma, cost, caregiving responsibilities, partners, etc.)
4. What other concerns have you heard from women about initiating a PrEP regimen?
5. Specific complaints (side effects, inconvenience, etc)?
6. Are there any aspects of the PrEP project that you’d like to comment on, which we haven’t already covered?

*Thank you for your time today.*
*We appreciate you sharing your perspective and feedback.*

*We also appreciate your important contributions to this initiative.*
